# The evaluation of prothrombin time and activated partial thromboplastin time among diabetic and healthy controls in Africa: systematic review and meta-analysis

**DOI:** 10.3389/fmed.2024.1445031

**Published:** 2024-12-06

**Authors:** Fasil Getu, Ermiyas Alemayehu, Addisu Tesfaye, Birhanu Genanew, Muluken Walle

**Affiliations:** ^1^Department of Medical Laboratory Science, College of Medicine and Health Sciences, Jigjiga University, Jigjiga, Ethiopia; ^2^Department of Medical Laboratory Science, College of Medicine and Health Sciences, Wollo University, Dessie, Ethiopia; ^3^Department of Medical Laboratory Science, College of Medicine and Health Sciences, Arba Minch University, Arba Minch, Ethiopia

**Keywords:** prothrombin time, activated partial thromboplastin time, diabetes mellitus, systematic review and meta-analysis, Africa

## Abstract

**Introduction:**

Diabetes Mellitus (DM) is a disorder of multiple etiologies characterized by chronic hyperglycemia resulting from defects in insulin secretion and/or insulin action. DM patients have a disturbance of hemostasis, leading to a prothrombotic state characterized by platelet hypersensitivity, coagulation factor disorders, and hypo-fibrinolysis. Therefore, the primary goal of this systematic review and meta-analysis was to determine the pooled Standard Mean Difference (SMD) of prothrombin time (PT) and activated partial thromboplastin time (APTT) of DM patients in Africa.

**Methods:**

This systematic review and meta-analysis was conducted based on the guidelines of the PRISMA. PubMed, Google Scholar, Science Direct, Dove Press, Cochrane Online, and African journals online were searched systematically. The qualities of the included studies were assessed by two independent reviewers using the JBI critical appraisal tools. Data were extracted in an Excel sheet and then exported to STATA version 11 for analysis. A Random-effect model was fitted to estimate the pooled SMD and Higgins I-square test statistics were done to test the heterogeneity of studies. Funnel plots analysis and Egger-weighted regression tests were done to detect publication bias.

**Results:**

The pooled SMD of PT among DM patients in Africa was −0.18, (95% CI: −0.72, 0.36). The pooled SMD of APTT among DM patients in Africa was −0.48, (95% CI: −1.18, 0.21). There was no statistically significant difference in the SMD of PT and APTT among DM patients in Africa compared to healthy controls. The pooled SMD of APTT among Type 1 DM patients in Africa was 0.86 (95% CI: 0.04, 1.69) whereas the SMD among Type 2 DM was −0.42 (95% CI: −1.24, 0.40). The SMD of APTT in Type 1 DM and controls showed a statistically significant difference compared with Type 2 DM and controls (*p* = 0.041). The SMD of APTT in Africa that is determined using a case–control study design showed a statistically significant difference compared to the SMD that is determined using a comparative cross-sectional study design.

**Conclusion and recommendations:**

Even though different studies conducted across African countries showed the presence of coagulation abnormality in DM, this systematic review and meta-analysis revealed that there is no statistically significant SMD of PT and APTT in DM patients compared to healthy controls. However it is recommended that physicians routinely check APTT levels in Type I DM patients in order to evaluate coagulation status.

## Introduction

Diabetes mellitus (DM) is a group of heterogeneous disorders of multiple etiologies characterized by chronic hyperglycemia resulting from defects in insulin secretion and/or insulin action (1). According to the current classification, there are two major types of DM: type 1 diabetes and type 2 diabetes (2). Type 2 diabetes is the most common type of diabetes, accounting for over 90% of all diabetes worldwide (3). According to the American diabetic association, patients have diabetes if, fasting serum glucose level ≥ 7.0 mmol/L (126 mg/dL), 2-h post-load (75 g) plasma glucose level ≥ 11.1 mmol/L (200 mg/dL), Hemoglobin A1c level ≥ 6.5% (48 mmol/mol) or a random blood glucose level ≥ 11.1 mmol/L (200 mg/ dl) in the presence of signs and symptoms. Two abnormal test results, either from the same sample or from two different test samples, are needed for diagnosis in the absence of unequivocal hyperglycemia (1).

International Diabetic Federation (IDF) 2021 data showed that, globally diabetes affects an estimated 537 million adults (20–79 years) and 24 million people in the Africa region. It is also predicted that the global prevalence will increase to 643 million by the year 2030, and 783 million by the year 2045. Approximately 6.7 million adults are estimated to have died as a result of diabetes, or its complications in 2021 (3). The highest prevalence (75%) was observed in low and middle-income countries. Globally, one in two people living with diabetes is undiagnosed (4). The majority, 70% of patients with undiagnosed diabetes are found in sub-Saharan Africa (5). The main abnormality among Africans from sub-Saharan Africa is hyperinsulinemia, which is brought on by a combination of decreased hepatic insulin clearance and increased insulin secretion. This condition exacerbates insulin resistance and obesity, which in turn causes beta cell failure and type 2 diabetes (6).

Coagulation and hemostasis involve interactions between tissue and coagulation factors as well as blood and endothelial cells, finally resulting in the formation of fibrin clots stopping bleeding (7). During this process, the fibrinolytic system decomposes generated clots to prohibit widespread thrombus formation and vascular occlusion (8). In patients having DM, metabolic disorders disturb these physiological mechanisms, leading to a prothrombotic state characterized by platelet hypersensitivity, coagulation factor disorders, and hypo-fibrinolysis (9). Both, quantitative and qualitative alterations of coagulation and anticoagulation factors were observed in patients with DM, contributing to the formation of lysis-resistant clots (10).

Coagulation abnormalities with a decreased level of antithrombin III, protein C, and protein S have been reported in DM with elevated clotting factors VII (11). Moreover, there is also an increase in plasminogen activator inhibitor type 1 which decreases fibrinolysis. Together they contribute to a hypercoagulable state in DM. Hypercoagulability in diabetes may accelerate atherosclerosis and act as a risk factor for the development of Cardiovascular Diseases (CVD). Hypercoagulability and endothelial dysfunction contribute significantly to the development of vascular problems in DM patients, including CVDs (12).

Measurements of Prothrombin Time (PT) and Activated Partial Thromboplastin Time (APTT) are usually done in patients with suspected coagulation abnormalities (13). PT and APTT are tests that quantify the activation of extrinsic and intrinsic coagulation pathways, respectively (14). The study holds paramount importance for both Africa and the global health community. Africa faces a mounting burden of diabetes mellitus, making it crucial to understand the associated complications, including alterations in coagulation parameters. Research on diabetes and coagulation profiles in African populations is limited, highlighting the significance of this study in filling critical knowledge gaps. By systematically reviewing and meta-analyzing available data, the study provides valuable insights into the patterns of altered coagulation parameters among diabetic individuals in Africa. Ultimately, these findings can inform clinical practice, aid in risk stratification, and guide preventive measures to mitigate the burden of cardiovascular diseases in diabetic populations, both regionally and globally. Moreover, there is no previously done systematic review and meta-analysis that estimates the pooled Standard Mean Difference (SMD) of PT and APTT among DM patients in Africa. Therefore, the primary goal of this systematic review and meta-analysis was to determine the pooled SMD of PT and APTT among DM patients and control groups in Africa.

## Methods

### Study protocol

This systematic review and meta-analysis was conducted following the guidelines of the Preferred Reporting Items for Systematic Review and Meta-Analysis (PRISMA) (15). To determine the pooled SMD of PT and APTT among DM patients and controls in Africa, findings from published articles have been used. This systematic review and meta-analysis was registered on the International Prospective Register of Systematic Reviews (CRD42023479650).

### Eligibility criteria

#### Inclusion criteria

Studies that are accepted and published in peer-reviewed journals were included. Participants in this study were from various racial and ethnic groups, socioeconomic classes, levels of education, and African countries. Comparative cross-sectional and case–control studies reporting the outcome of interest were included. This systematic review and meta-analysis included all studies that were published before September 2023. Database searching was conducted on February 29, 2024. Articles that were published and available as open access and written in English language were eligible for inclusion. Results that were expressed as median and interquartile range were also extracted and changed to mean and Standard Deviation (SD) using an Excel sheet that contains the statistical formula.

#### Exclusion criteria

Following a careful review of the entire texts and abstracts, the following research was not included

- Studies that did not report the overall Mean Differences (MD) and SD of PT and APTT
- Studies that were case reports, reviews, poster presentations, and letters to the editor; and
- Studies which were published in non-English languages

#### Study outcome

The outcome variables in this systematic review and meta-analysis were MD and SD of PT and APTT among DM patients and health controls.

### Information sources

By searching for previously published literature, data were acquired from sources like PubMed, Google Scholar, Science Direct, Cochrane Online, Embase, SCOPUS, and African journals online.

### Searching strategy

We have conducted a wide-ranging search of eligible studies in PubMed, Google Scholar, Science Direct, Cochrane Online, Embase, SCOPUS, and African journals online. Reference probing of identified articles was performed to identify additional relevant studies. The search strategy was based on the combinations of keywords and Medical Subject Heading (MeSH) terms. They were used separately and in combination using Boolean operators like “OR” or “AND.” The search terms used in electronic databases were “Prothrombin time,” “Activated partial thromboplastin time,” “Coagulation parameters,” “Coagulation profile,” “Hemostatic profile,” and “Diabetes mellitus.” Additional filters such as language (English) and study population (Human) were used.

### Study selection and quality assessment

To gather and arrange search results and to filter out duplicate articles, the saved articles were imported into EndNote X9. The articles were then individually reviewed by two reviewers by reading their titles and abstracts. During the review process, two reviewers had a lively discussion and reached an agreement. If there were any disputes, a third reviewer was brought in. Using the critical appraisal tools developed by the Joanna Briggs Institute (JBI), the reviewers evaluated the methodological quality of the included studies (16). The tools include questions to rate internal and external validity. Different JBI critical appraisal tools were used to evaluate research that used different study designs. Each item was carefully used to assess the methodological quality of the included studies. A value of one and zero was given for each research according to JBI critical appraisal tools. For items that are clearly stated in the method, a value of one (1) was assigned, while for items that are not clearly stated in the method part of the research, a value of zero (0) was assigned. Finally, a percentage representing the overall methodological quality of the included studies was calculated. Articles with methodical quality of less than 50%, more than 50%, and greater than 75% were rated as poor, good, and high quality, respectively (17).

### Data extraction

After evaluating the methodological quality, Studies that met the eligibility requirements were subjected to data extraction by all reviewers using a prepared data extraction sheet. The following items were extracted for analysis: Name of the first author, study area, study region, study design, year of publication, sample size, type of DM, mean and SD of PT, and mean and SD of APTT (Table 1).

**Table 1:** Summary of included studies in this systematic review and meta-analysis.

Authors	Study area	Region	Study design	Year	Type of DM	Sample size	PT Mean ± SD	APTT Mean ± SD
Ebrahim et al. (22)	Ethiopia	East	Comparative cross-sectional	2021	Type 1	Case = 60	13.2 ± 2.9	24.4 ± 5.3
						Control = 60	13.9 ± 1.7	23.4 ± 5.1
					Type 2	Case = 60	12.5 ± 2.9	23.1 ± 4
						Control = 60	13.9 ± 1.7	23.4 ± 5.1
Abdeen and Hamza (26)	Sudan	East	Case–control	2014	Type 1	Case = 14	14.64 ± 1.8	30.40 ± 5.2
						Control = 20	14.14 ± 0.521	25.95 ± 3.09
					Type 2	Case = 86	14.17 ± 1.05	26.51 ± 3.4
						Control = 20	14.14 ± 0.521	25.95 ± 3.09
Fattah et al. (21)	Egypt	North	Comparative cross-sectional	2003	Type 1	Case = 45	12.31 ± 0.22	36.87 ± 1.3
						Control = 45	12.85 ± 0.29	35.48 ± 0.63
					Type 2	Case = 45	13.3 ± 0.34	36.29 ± 0.55
						Control = 45	12.73 ± 0.32	35.16 ± 0.71
Ambelu et al. (27)	Ethiopia	East	Comparative cross-sectional	2018	Type 2	Case = 40	14.65 ± 2.5	34.4 ± 5.35
						Control = 40	14.28 ± 1.5	32.79 ± 4.12
Ephraim et al. (25)	Ghana	West	Case–control	2017	Type2	Case = 60	11.03 ± 2.06	20.88 ± 5.19
						Control = 40	14.46 ± 1.86	31.23 ± 5.41
Ukamaka et al. (28)	Nigeria	West	Comparative cross-sectional	2019	Type 2	Case = 150	13.47 ± 1.25	34.39 ± 2.17
						Control = 150	14.06 ± 0.79	37.25 ± 2.1
Omer (29)	Sudan	East	Comparative cross-sectional	2019	Non-classified	Case = 57	16.091 ± 3.06	44.979 ± 25.47
						Control = 20	14.57 ± 1.32	34.32 ± 3.62
Bashir and Ali (30)	Sudan	East	Case–control	2018	Type 2	Case = 57	12.61 ± 2.57	32.64 ± 5.2
						Control = 57	13.67 ± 1.59	28.49 ± 4.13
Mohammed (31)	Sudan	East	Case–control	2016	Type 2	Case = 60	13.6 ± 1.52	24.9 ± 2.794
						Control = 30	13.57 ± 1.478	38.17 ± 4.594
Boshabor (32)	Libya	North	Case–control	2022	Non-classified	Case = 49	15.1905 ± 2.61952	29.76 ± 2.879
						Control = 21	15.4286 ± 3.2914	28.69 ± 3.293
Adejumo et al. (33)	Nigeria	West	Case–control	2023	Non-classified	Case = 215	12.64 ± 0.46	25.13 ± 0.44
						Control = 65	14.51 ± 0.12	29.84 ± 0.22
Elhassade and Balha (34)	Libya	North	Case–control	2016	Type 2	Case = 30	14.04 ± 2.96	28.95 ± 7.54
						Control = 20	13.5 ± 1.54	34.12 ± 2.82
Abdulrahaman and Dallatu (35)	Nigeria	West	Case–control	2016	Non-classified	Case = 50	16.72 ± 2.339	43.26 ± 5.587
						Control = 50	14.92 ± 1.209	41.38 ± 4.295
Asrat et al. (36)	Ethiopia	East	Comparative cross-sectional	2019	Non-classified	Case = 119	13.83 ± 1.65	30.6 ± 9.6
						Control = 119	13.53 ± 1.05	35.8 ± 5.7
Fadairo et al. (37)	Nigeria	West	Comparative cross-sectional	2016	Non-classified	Case = 50	15.26 ± 0.46	49.59 ± 3.06
						Control = 50	14.5 ± 0.3	45.35 ± 2.78
Alao et al. (38)	Nigeria	West	Comparative cross-sectional	2009	Type 2	Case = 50	15.7 ± 2.1	27.3 ± 4.4
						Control = 50	14.9 ± 2.3	25.3 ± 3.9
Ifeanyi et al. (39)	Nigeria	West	Comparative cross-sectional	2014	Non-classified	Case = 50	17.2 ± 3.2	29.6 ± 2.7
						Control = 50	16.5 ± 2.8	27.1 ± 3

### Statistical analysis

The data analysis tool of choice was STATA version 11. After entering the data into Excel, it was exported to STATA for additional analysis. Random-effect model meta-analysis was used to estimate the pooled effect size and effect of each study with their confidence interval. Higgin I-Square statistics was used to measure the degree of heterogeneity between the included studies in the meta-analysis (18). If the I-Square value was 25, 50, and 75% they were assumed to show low, medium, and high heterogeneity, respectively. Sub-group analysis and sensitivity analyses were employed to resolve the occurrence of high heterogeneity in the included studies. Funnel plots analysis and Egger weighted regression test were done to detect publication bias. A *p* value of <0.05 in Egger’s test was considered evidence of statistically significant publication bias (19).

### Ethics approval

Since this study does not involve any human or animal participants, ethical approval was not requested for it.

## Results

### Literature search and identified results

A total of 5,504 studies were identified through a database literature search including manual

**Figure 1:**
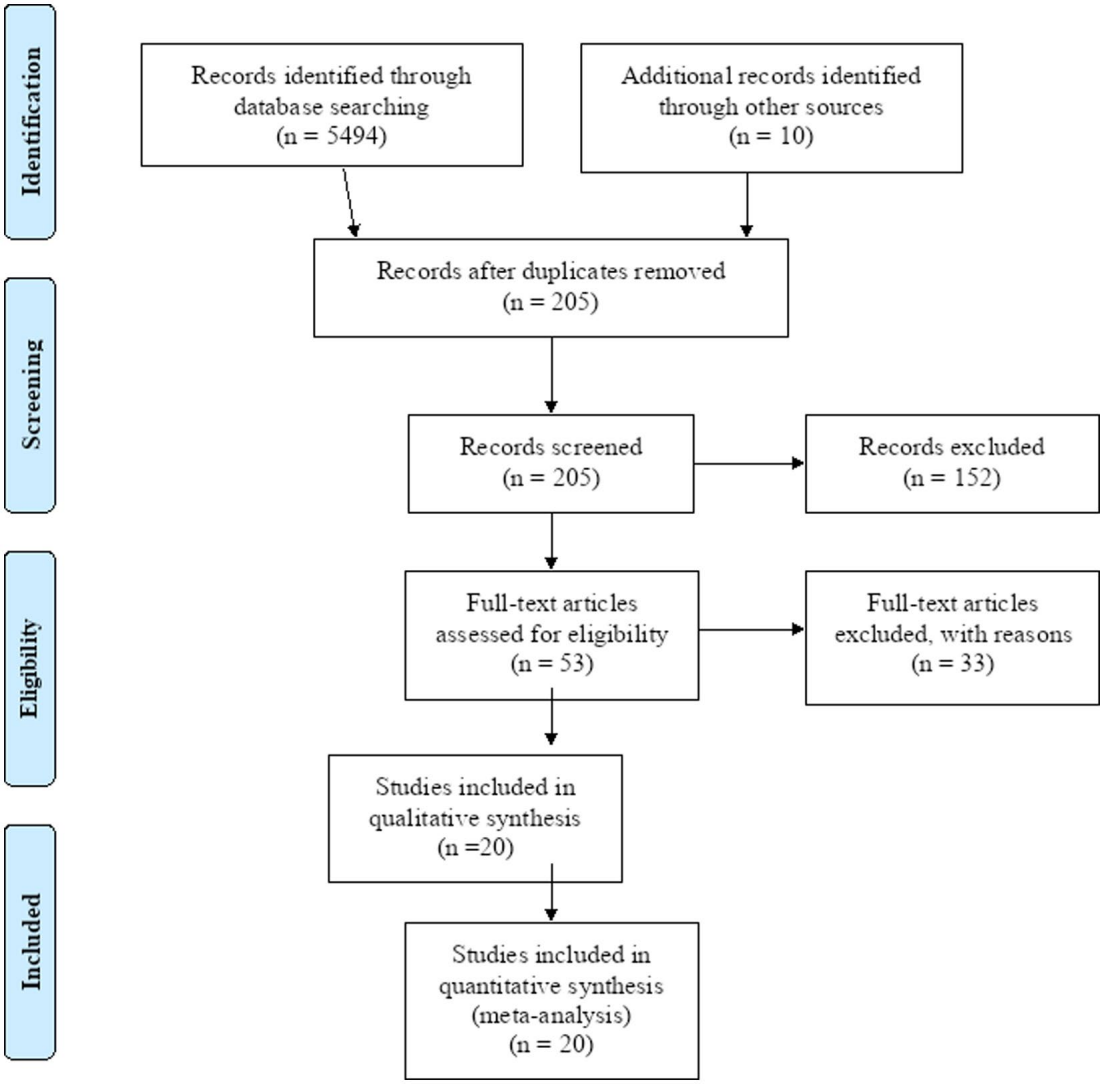
Flow chart of the selection of studies for the systematic review and meta-analysis on the evaluation of prothrombin time and activated partial thromboplastin time among diabetic and healthy controls in Africa.

Search. After the removal of duplicates and irrelevant studies, we got a total of 205 studies. Then 205 articles were screened. Out of them, 152 studies were removed by reading their titles. From the remaining 53 studies, 33 studies were removed because of the dissimilarity of the study area. Finally, after excluding irrelevant articles, 20 full-text were identified and used for the final qualitative and quantitative analysis (Figure 1).

### Study characteristics

In this systematic review and meta-analysis, 20 articles were included. Out of them, 9 articles were included from East Africa, 4 from North Africa, and 7 were from West Africa. The sample size of the included study ranged from 14 to 215 DM patients. Of the total included studies, 12 of them were comparative cross-sectional studies whereas, 8 of them were case–control studies (Table 1). The methodological quality of the final included studies was high. For comparative cross-sectional studies, the range of the quality score was 6–9 (mean = 7.5). For case control studies, the range of the quality score was 7–10 (mean = 8.5).

### The pooled standard mean difference of prothrombin time among diabetes patients compared to healthy controls in Africa

**Figure 2:**
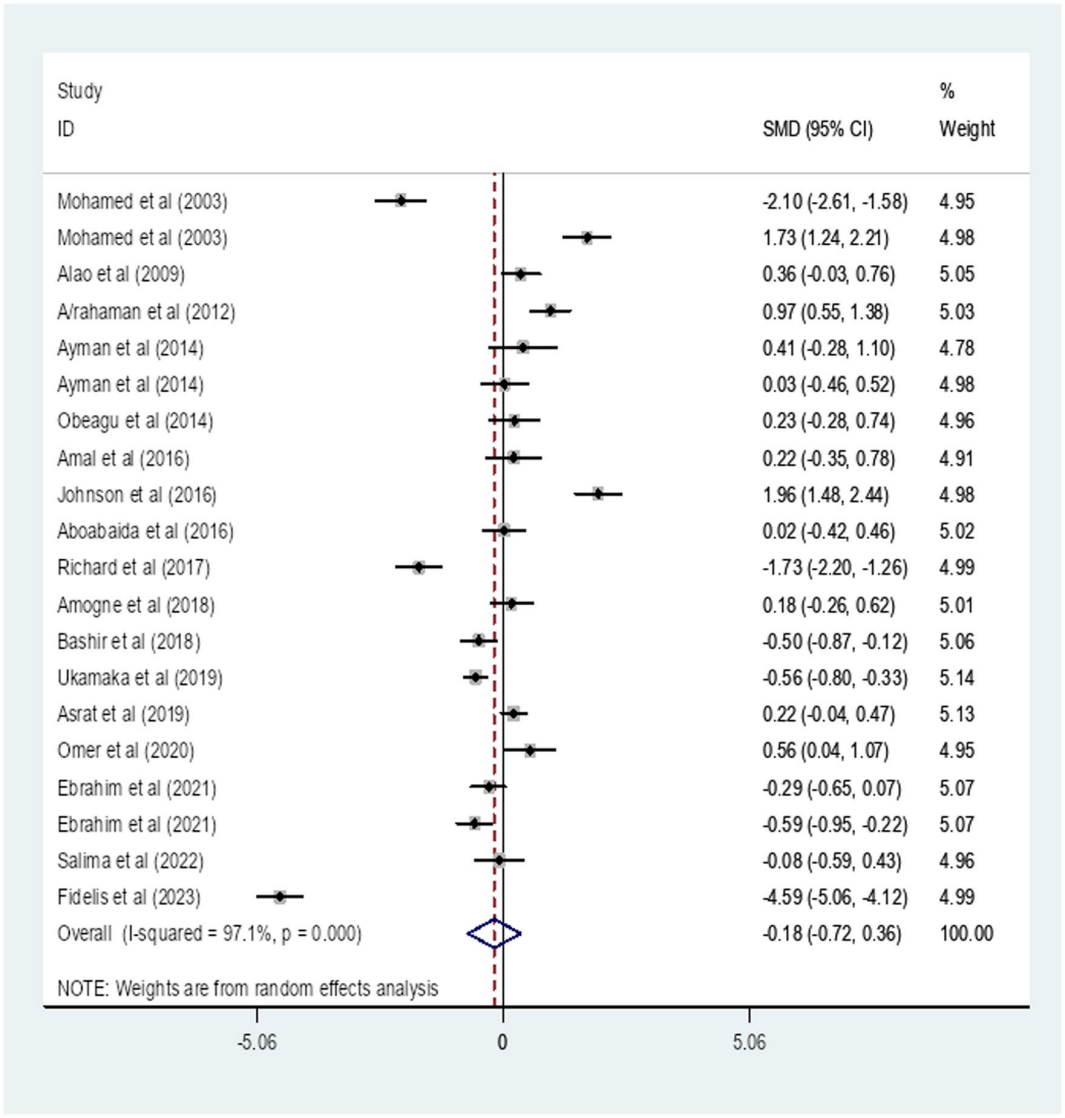
The pooled SMD of PT among diabetes patients in Africa. SMD, Standard Mean Difference; I-square, heterogeneity statistic.

We performed a random-effect meta-analysis of pooled SMD for PT on the extracted 20 studies. The overall pooled SMD of PT among diabetes patients in Africa was −0.18, (95% CI: −0.72, 0.36) with an I-square value of 97.1% (*p* = 0.514) (Figure 2).

### Subgroup analysis of the pooled standard mean difference of prothrombin time among diabetes patients compared to healthy controls in Africa based on the type of diabetes

**Figure 3:**
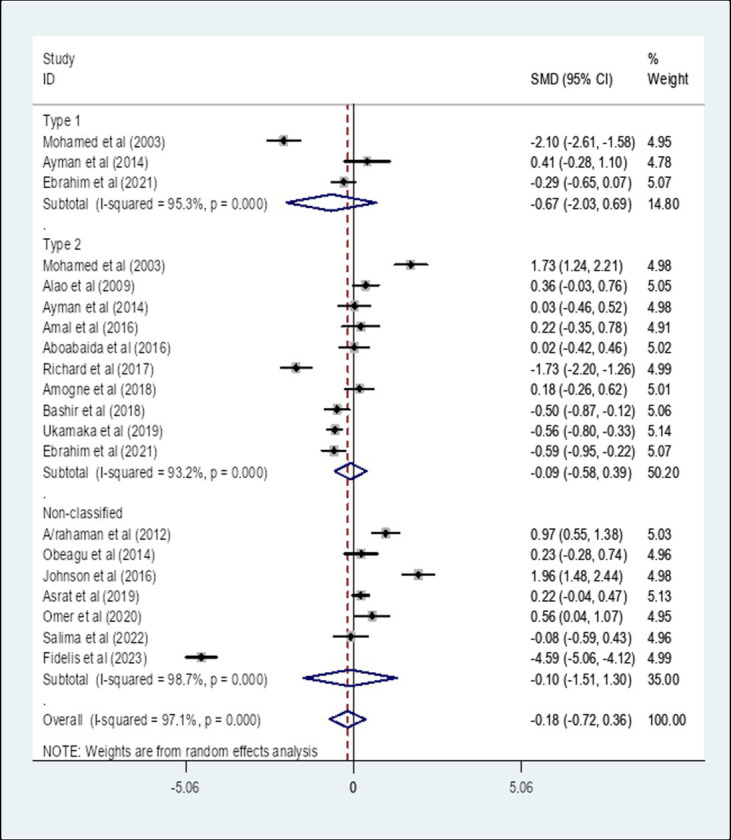
The pooled SMD of PT among diabetes patients in Africa based on the type of diabetes. SMD, Standard Mean Difference; I-square, heterogeneity statistic.

A subgroup analysis by the type of diabetes showed that the pooled SMD of PT among Type 1 diabetes patients in Africa was −0.67 (95% CI: −2.03, 0.69) with an I-square value of 95.3% (*p* = 0.333) whereas the SMD among Type 2 diabetes was −0.09 (95% CI: −0.58, 0.39) with an I-square value of 93.2% (*p* = 0.710). The SMD of PT among unclassified diabetes was −0.10 (95% CI: −1.51, 1.30) with an I-square value of 98.7% (*p* = 0.884) (Figure 3).

### Subgroup analysis of the pooled standard mean difference of prothrombin time among diabetes patients compared to healthy controls in Africa based on region

**Figure 4:**
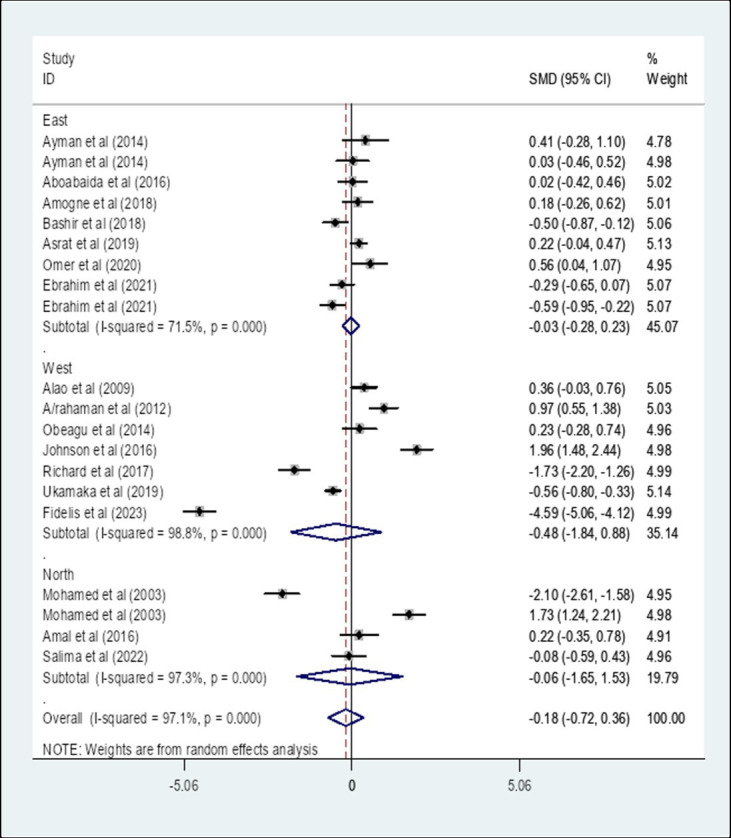
The pooled SMD of PT among diabetes patients in Africa based on region. SMD, Standard Mean Difference; I-square, heterogeneity statistic.

A subgroup analysis by region showed that the pooled SMD of PT among diabetes patients in East Africa was −0.03 (95% CI: −0.28, 0.23) with an I-square value of 71.5% (*p* = 0.833) whereas the SMD among diabetes patients in West Africa was −0.48 (95% CI: −1.84, 0.88) with an I-square value of 98.8% (*p* = 0.490). The SMD of PT among diabetes in North Africa was −0.06 (95% CI: −1.65, 1.53) with an I-square value of 97.3% (*p* = 0.943) (Figure 4).

### Subgroup analysis of the pooled standard mean difference of prothrombin time among diabetes patients compared to healthy controls in Africa based on the study design

**Figure 5:**
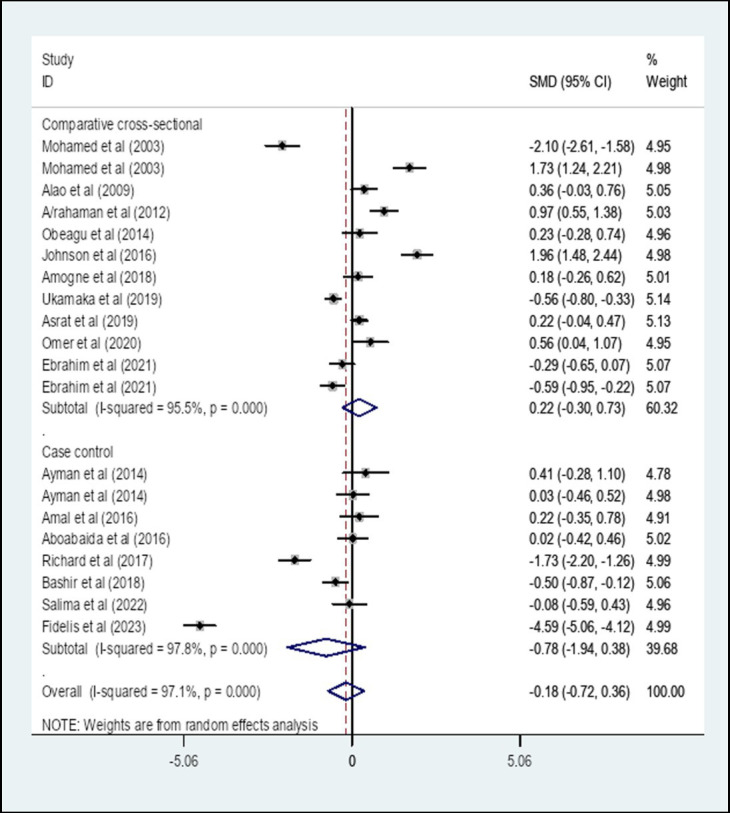
The pooled SMD of PT among diabetes patients in Africa based on study design. SMD, Standard Mean Difference; I-square, heterogeneity statistic.

A subgroup analysis by study design showed that the pooled SMD of PT among diabetes patients in Africa that is determined using comparative cross-sectional study design was 0.22 (95% CI: −0.30, 0.73) with an I-square value of 95.5% (*p* = 0.411) whereas the SMD using case–control study design was −0.78 (95% CI: −1.94, 0.38) with an I-square value of 97.8% (*p* = 0.186) (Figure 5).

### Heterogeneity and publication bias

**Figure 6:**
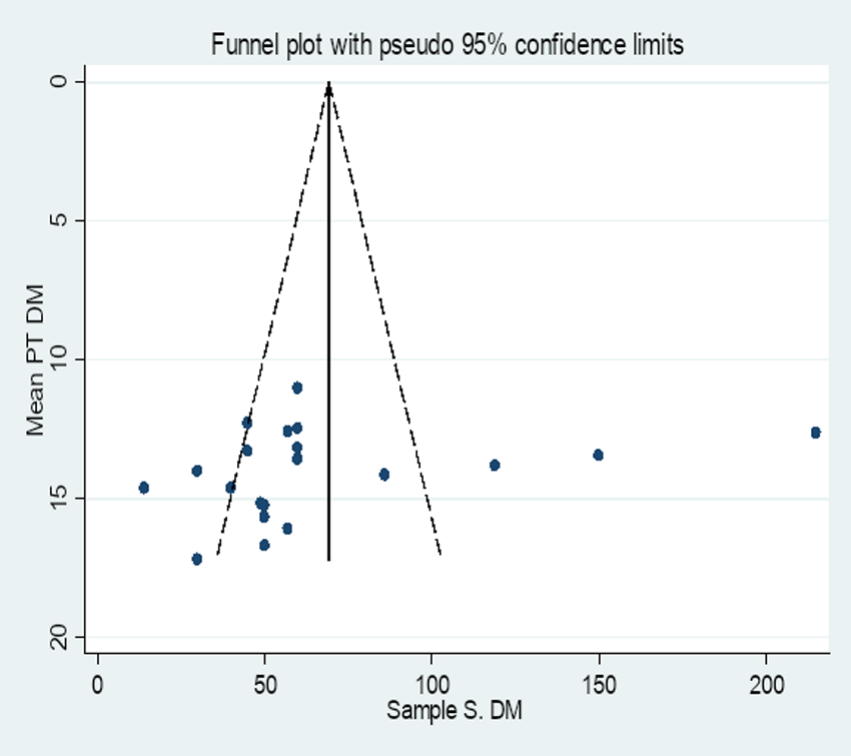
Funnel plot of the included studies to determine the pooled SMD of PT among diabetes patients in Africa.

The included studies were assessed for potential publication bias visually by funnel plot and Egger’s statistics. In this review the funnel plot of the included studies is asymmetric (Figure 6). In addition, the Egger weighted regression statistics showed that (*p* < 0.05) (in this case *p* = 0.002), indicating that there is publication bias (Table 2).

**Table 2:** Egger’s test of the included studies for the determination of the pooled SMD of PT among diabetic patients in Africa.

Std_Eff	Coef.	Std. Err.	*t*	P > t	[95% CI]	
Slope	−2.568076	4.70956	−0.55	0.592	−12.46249	7.326342
Bias	1.174051	0.3332655	3.52	0.002	0.4738858	1.874216

### Sensitivity analysis

**Figure 7:**
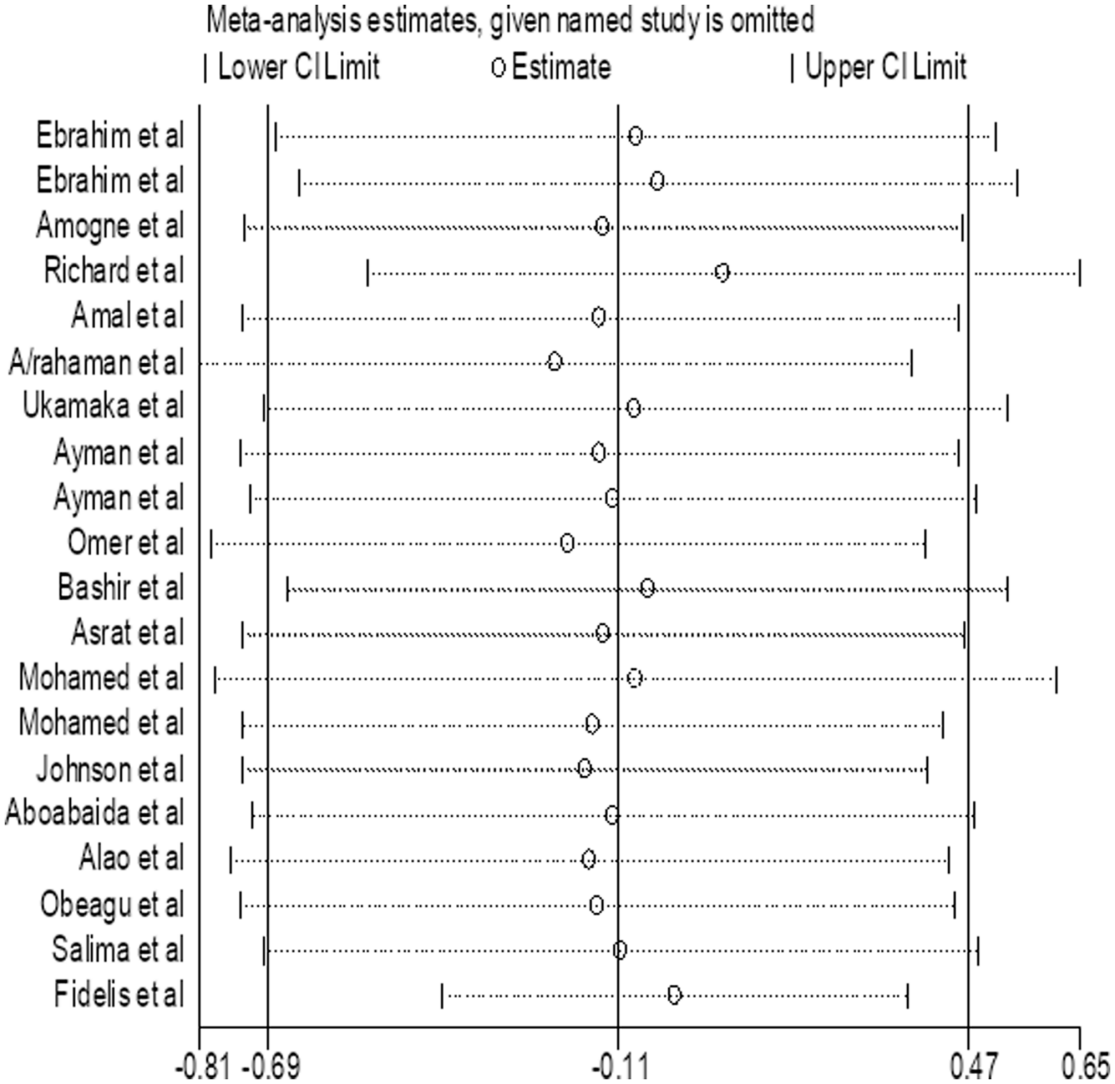
Forest plot of the included studies to determine the pooled SMD of PT among diabetes patients in Africa.

A sensitivity analysis was carried out by applying random effect models. The analysis was done to evaluate the influence of each study on the pooled estimated SMD of PT among DM patients. The result showed that omitted studies did not show a significant effect on the pooled SMD of PT among DM patients in Africa (Figure 7).

### The pooled standard mean difference of activated partial thromboplastin time among diabetes patients compared to healthy controls in Africa

**Figure 8:**
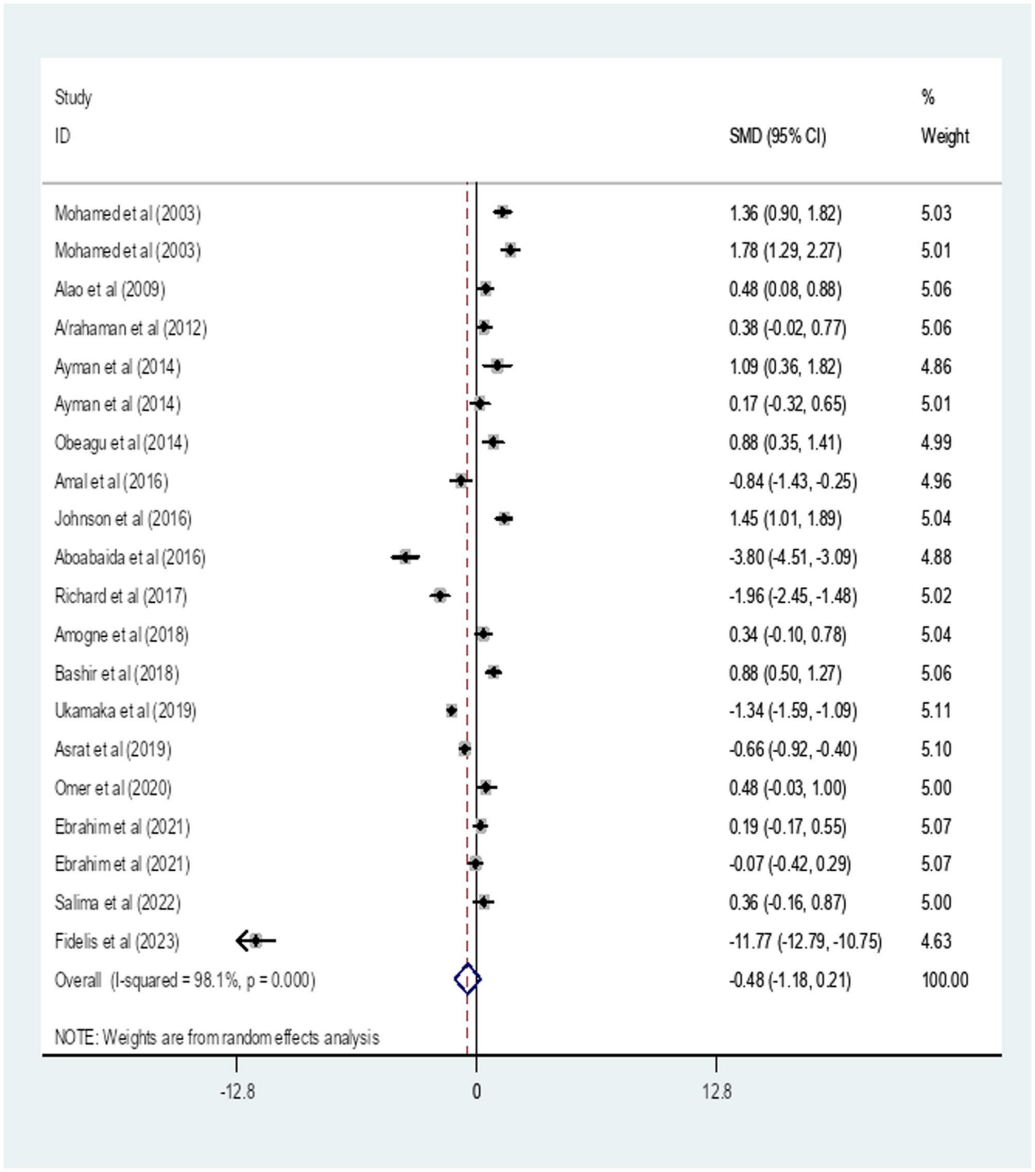
The pooled SMD of APTT among diabetes patients in Africa. SMD, Standard Mean Difference; I-square, heterogeneity statistic.

We performed a random-effect meta-analysis of pooled SMD for APTT on the extracted 20 studies. The overall pooled SMD of APTT among diabetes patients in Africa was −0.48, (95% CI: −1.18, 0.21) with an I-square value of 98.1% (*p* = 0.174) (Figure 8).

### Subgroup analysis of the pooled standard mean difference of activated partial thromboplastin time among diabetes patients compared to healthy controls in Africa based on the type of diabetes

**Figure 9:**
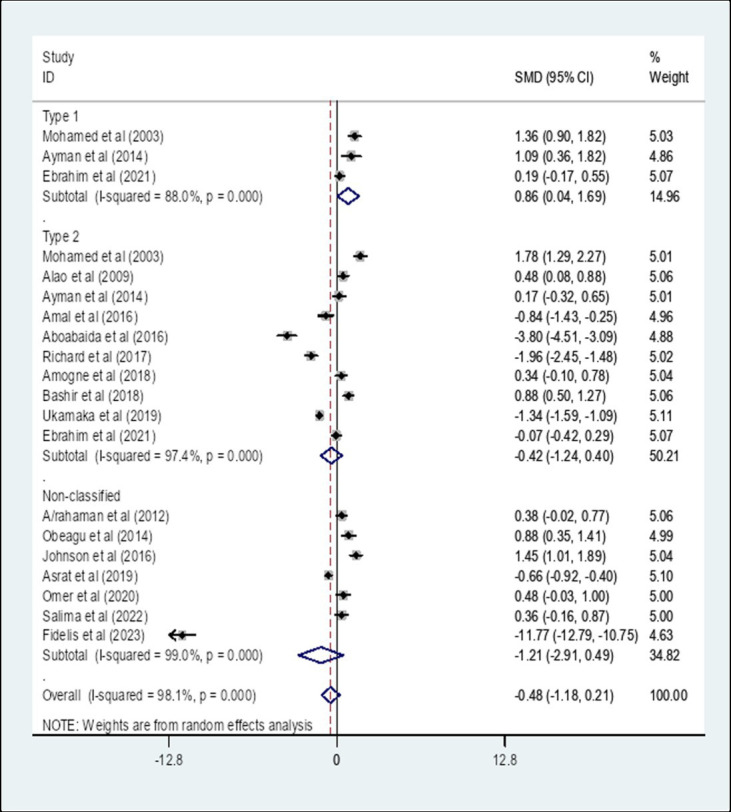
The pooled SMD of APTT among diabetes patients in Africa based on the type of diabetes. SMD, Standard Mean Difference; I-square, heterogeneity statistic.

A subgroup analysis by the type of diabetes showed that the pooled SMD of APTT among Type 1 diabetes patients in Africa was 0.86 (95% CI: 0.04, 1.69) with an I-square value of 88% (*p* = 0.041) whereas the SMD among Type 2 diabetes was −0.42 (95% CI: −1.24, 0.40) with an I-square value of 97.4% (*p* = 0.313). The SMD of APTT among unclassified diabetes was −1.21 (95% CI: −2.91, 0.49) with an I-square value of 98.7% (*p* = 0.162) (Figure 9).

### Subgroup analysis of the pooled standard mean difference of activated partial time among diabetes patients compared to healthy controls in Africa based on region

**Figure 10:**
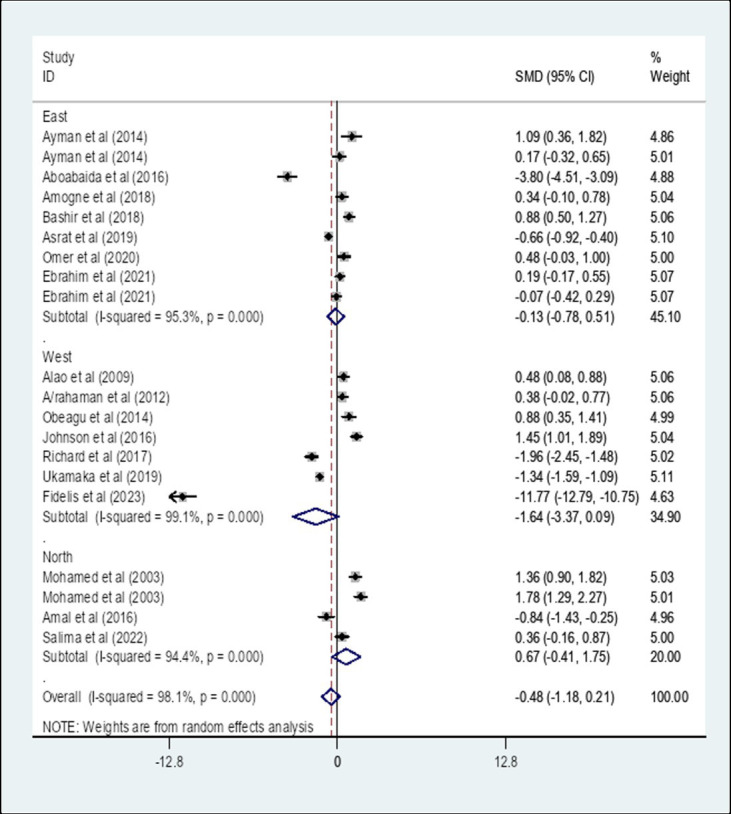
The pooled SMD of APTT among diabetes patients in Africa based on region. SMD, Standard Mean Difference; I-square, heterogeneity statistic.

A subgroup analysis by region showed that the pooled SMD of APTT among diabetes patients in East Africa was −0.13 (95% CI: −0.78, 0.51) with an I-square value of 95.3% (*p* = 0.833) whereas the SMD among diabetes patients in West Africa was −1.64 (95% CI: −3.37, 0.09) with an I-square value of 99.1% (*p* = 0.062). The SMD of APTT among diabetes in North Africa was 0.67 (95% CI: −0.41, 1.75) with an I-square value of 94.4% (*p* = 0.222) (Figure 10).

### Subgroup analysis of the pooled standard mean difference of activated partial thromboplastin time among diabetes patients compared to healthy controls in Africa based on the study design

**Figure 11:**
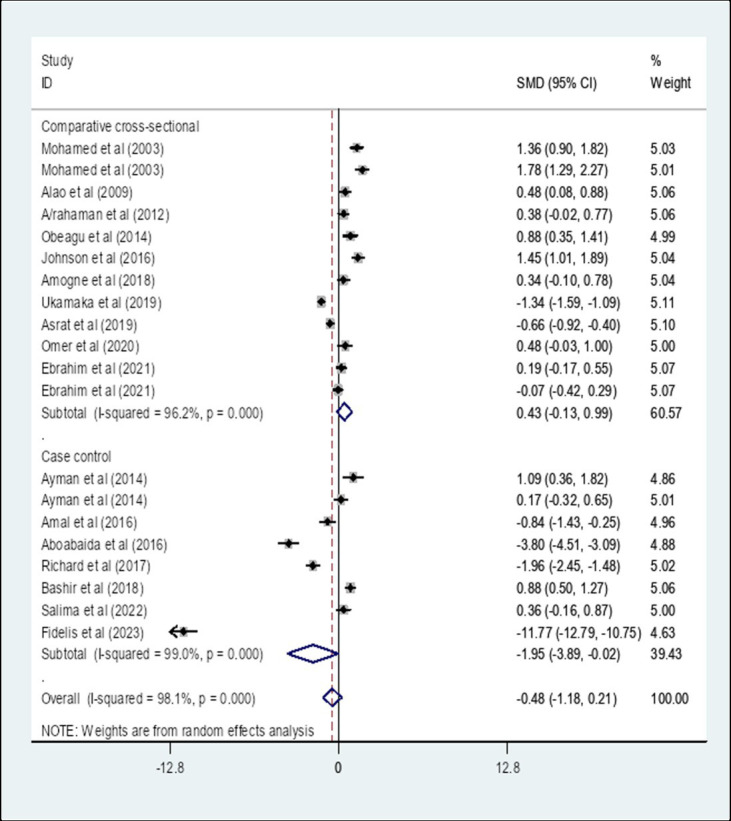
The pooled SMD of APTT among diabetes patients in Africa based on study design. SMD, Standard Mean Difference; I-square, heterogeneity statistic.

A subgroup analysis by study design showed that the pooled SMD of APTT among diabetes patients in Africa that is determined using a comparative cross-sectional study design was 0.43 (95% CI: −0.13, 0.99) with an I-square value of 96.2% (*p* = 0.136) whereas the SMD using case–control study design was −1.95 (95% CI: −3.89, −0.02) with an I-square value of 99% (*p* = 0.048) (Figure 11).

### Heterogeneity and publication bias

**Figure 12:**
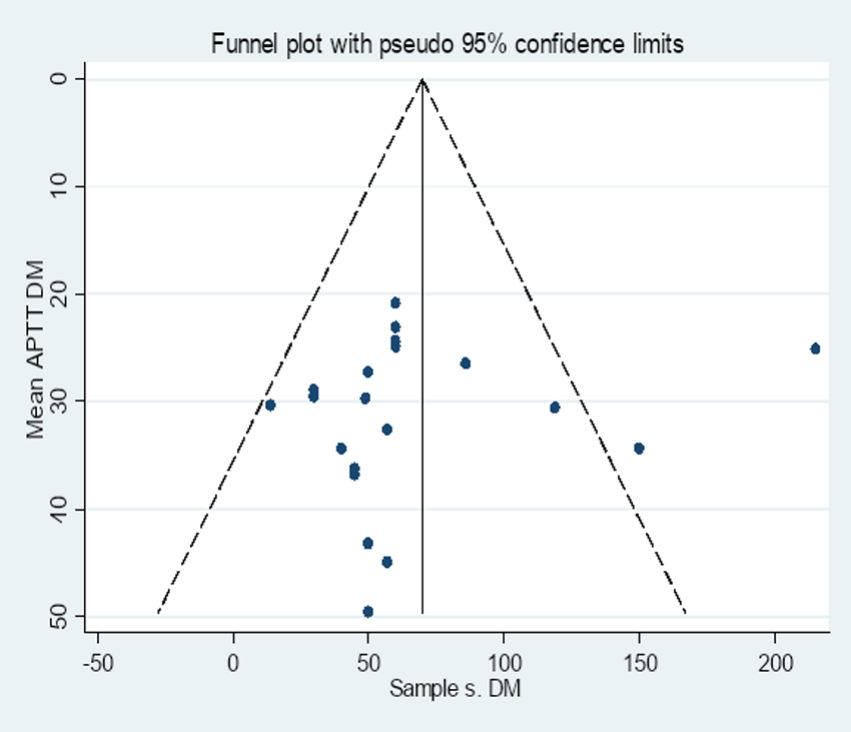
Funnel plot of the included studies to determine the pooled SMD of APTT among diabetes patients in Africa.

The included studies were assessed for potential publication bias visually by funnel plot and Egger’s statistics. In this review the funnel plot of the included studies is asymmetric (Figure 12). In addition, the Egger weighted regression statistics showed that (*p* < 0.05) (in this case *p* = 0.001), indicating that there is publication bias (Table 3).

**Table 3:** Egger’s test of the included studies for the determination of the pooled SMD of APTT among DM patients in Africa.

Std_Eff	Coef.	Std. Err.	*t*	P > t	[95% CI]	
Slope	5.551142	6.094726	0.91	0.374	7.253401	18.35569
Bias	0.8171948	0.2004815	4.08	0.001	0.3959987	1.238391

### Sensitivity analysis

**Figure 13:**
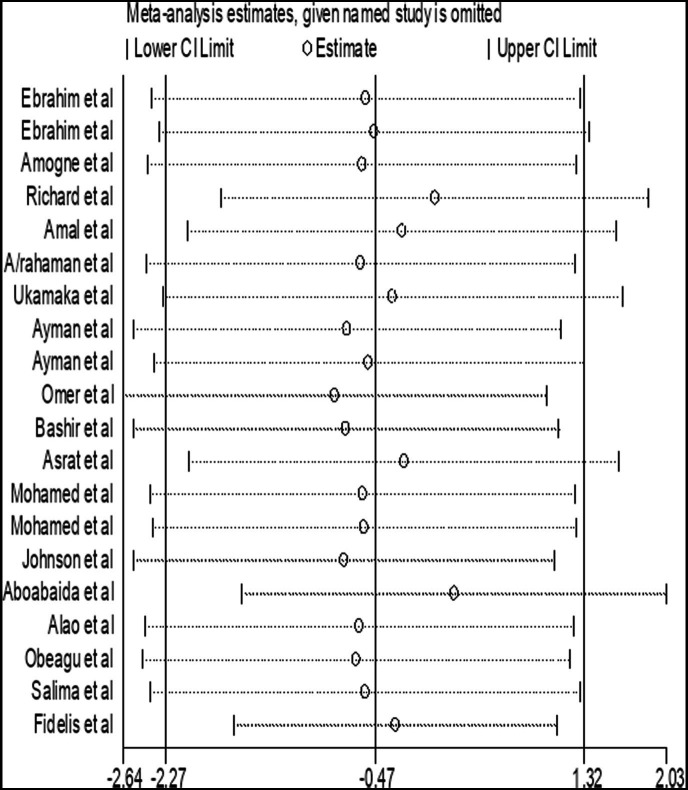
Forest plot of the included studies to determine the pooled SMD of APTT among diabetes patients in Africa.

A sensitivity analysis was carried out by applying random effect models. The analysis was done to evaluate the influence of each study on the pooled estimated SMD of APTT among DM patients. The result showed that omitted studies did not show a significant effect on the pooled SMD of APTT among DM patients (Figure 13).

## Discussion

Diabetes is a procoagulant state (20). The primary cause of morbidity and death in diabetic patients is atherothrombotic disease, which is typically linked to both vascular and metabolic abnormalities (21). DM is associated with a significant increase in the risk of cardiovascular disease by activating coagulation factors, increasing platelet activation, and hypofibrinolysis (22). In patients with diabetes mellitus, hypercoagulability and endothelial dysfunction play a major role in the development of vascular issues, including CVD (12). This systematic review and meta-analysis was conducted to determine the pooled SMD of PT and APTT among DM patients in Africa using published articles. The MD varies from study to study in different African countries. This variation could result from variations in the included studies’ sample sizes, research designs, participant socioeconomic characteristics, and the type of coagulation analyzer used in the included articles (22).

The overall pooled SMD of PT among DM patients in Africa was −0.18, (95% CI: −0.72, 0.36). This indicated that PT is slightly decreased among DM patients compared to healthy controls in Africa, but not significant (*p* = 0.514). This could occur as a result of the glycation of intrinsic and extrinsic clotting factors in DM patients who have prolonged hyperglycemia (23). Since a significant heterogeneity was found, a subgroup analysis by the type of DM was performed. The result showed that the pooled SMD of PT among Type 1 DM patients in Africa was −0.67 (95% CI: −2.03, 0.69) whereas the SMD among Type 2 DM patients was −0.09 (95% CI: −0.58, 0.39). The SMD of PT among unclassified DM was −0.10 (95% CI: −1.51, 1.30). The result of the subgroup analysis by type of DM indicated that there is no statistically significant difference in the SMD of PT among Type 1 and Type 2 DM patients in Africa compared to their respective controls. This finding is in line with a study conducted in Egypt (21) that showed PT between Type 2 DM patients and controls had no significant difference. However, this result was in contradiction with the findings reported in Ethiopia (22), India (11), and Nigeria (24) which reported a significantly reduced PT in Type 2 DM patients. This variation might be due to the inclusion of studies conducted in different African countries.

Moreover, A subgroup analysis by a region was performed. The result showed that the pooled SMD of PT among DM patients in East Africa was −0.03 (95% CI: −0.28, 0.23) whereas the SMD among DM patients in West Africa was −0.48 (95% CI: −1.84, 0.88). The SMD of PT among DM patients in North Africa was −0.06 (95% CI: −1.65, 1.53). There was no statistically significant value SMD of PT in different African regions. Furthermore, A subgroup analysis by study design showed that the pooled SMD of PT among DM patients in Africa that is determined using a comparative cross-sectional study design was 0.22 (95% CI: −0.30, 0.73) whereas the SMD of PT using a case–control study design was −0.78 (95% CI: −1.94, 0.38). There is no statistically significant difference in SMD of PT that is determined using a comparative cross-sectional study design and case–control study design.

The overall pooled SMD of APTT among DM patients in Africa was −0.48, (95% CI: −1.18, 0.21) with an I-square value of 98.1%. The SMD of APTT among diabetes patients in Africa was insignificant (*p* = 0.174). Since a significant heterogeneity was found, a subgroup analysis by the type of DM was performed. A subgroup analysis by the type of DM showed that the pooled SMD of APTT among Type 1 DM patients in Africa was 0.86 (95% CI: 0.04, 1.69) with an I-square value of 88% (*p* = 0.041) whereas the SMD among Type 2 DM was −0.42 (95% CI: −1.24, 0.40) with an I-square value of 97.4% (*p* = 0.313). The SMD of APTT in Type 1 DM patients and controls showed a statistically significant difference compared with Type 2 DM and controls (p = 0.041). The possible reason can be the duration of the disease is relatively long in T1DM compared to T2DM. The relatively persistent hyperglycemia in type 1 DM may cause coagulopathies glycation of hemoglobin, prothrombin, fibrinogen, and other proteins involved in the clotting mechanism (25). The SMD of APTT among unclassified DM was −1.21 (95% CI: −2.91, 0.49).

Moreover, a subgroup analysis by region showed that the pooled SMD of APTT among DM patients in East Africa was −0.13 (95% CI: −0.78, 0.51) whereas the SMD among DM patients in West Africa was −1.64 (95% CI: −3.37, 0.09). The SMD of APTT among DM patients in North Africa was 0.67 (95% CI: −0.41, 1.75). There was no statistically significant SMD of APTT in different African regions. Furthermore, a subgroup analysis by study design showed that the pooled SMD of APTT among DM patients in Africa that is determined using a comparative cross-sectional study design was 0.43 (95% CI: −0.13, 0.99) (*p* = 0.136) whereas the SMD using case–control study design was −1.95 (95% CI: −3.89, −0.02) (*p* = 0.048). The SMD of APTT in Africa that is determined using a case–control study design showed a statistically significant difference compared to comparative cross-sectional studies. The possible variation might be due to the nature of the study designs. The case–control study measures events retrospectively whereas cross-sectional studies measure events prospectively.

## Strengths and limitations

The inclusive search across multiple databases, the use of diverse searching techniques, the critical evaluation of the included studies’ methodological quality through the use of JBI critical appraisal tools, and the application of PRISMA guidelines are among the strong points of this systematic review and meta-analysis. The limitations of this systematic review and meta-analysis were, that the representativeness of the results may also be impacted by the inclusion of studies that are only published in English. This review is limited by the study designs of the included studies, which were solely observational. The results of this review cannot be generalized to Africa as there are considerable gaps in data as all countries were not included in this review. The review is limited by the quality of the included studies. The other significant drawback of this systematic review and meta-analysis was the high level of heterogeneity that persisted in all of the analyses, even after we conducted subgroup analysis.

## Conclusion and recommendations

Even though different studies conducted across African countries showed the presence of coagulation abnormality in DM patients, this systematic review and meta-analysis revealed that there is no statistically significant SMD of PT and APTT in DM patients compared to healthy individuals. It is recommended that physicians routinely check APTT levels in Type I DM patients in order to evaluate coagulation status because a significant APTT finding is seen in these patients when compared to controls. Early detection of coagulation disorders may lower the risk of thrombotic events and enable prompt therapies. Additionally, improving overall patient care for the African population can be achieved by integrating APTT testing into routine diabetic management.

## Data Availability

The original contributions presented in the study are included in the article/Supplementary material, further inquiries can be directed to the corresponding author.
