# Supplementary material for: The evaluation of prothrombin time and activated partial thromboplastin time among diabetic and healthy controls in Africa: systematic review and meta-analysis
Source: Front Med (Lausanne). 2024 Dec 6;11:1445031. doi: 10.3389/fmed.2024.1445031 (PMC11658991; doi:10.3389/fmed.2024.1445031)
Supplement: Supplementary file 1 [file Data_Sheet_1.docx]

| **SN** | **Database** | **Search terms** | **Articles** | **date** |
| --- | --- | --- | --- | --- |
| 1 | Scopus | (TITLE-ABS-KEY (“Prothrombin time”) OR TITLE-ABS-KEY ("Activated partial thromboplastin time") OR TITLE-ABS-KEY ("Coagulation parameters") OR TITLE-ABS-KEY ("Coagulation profile") OR TITLE-ABS-KEY (" Hemostatic profile") AND TITLE-ABS-KEY (“Diabetes mellitus”) | 2497 | 31-08-2023 |
| 2 | Embase | ('Prothrombin time':ti,ab,kw OR 'Activated partial thromboplastin time':ti,ab,kw OR 'Coagulation parameters':ti,ab,kw OR 'Coagulation profile':ti,ab,kw OR ' Hemostatic profile':ti,ab,kw) AND ('Diabetes mellitus':ti,ab,kw) | 486 | 31-08-2023 |
| 3 | Pubmed | (“Prothrombin time" OR "Activated partial thromboplastin time" OR " Coagulation parameters" OR “Coagulation profile" OR "Hemostatic profile") AND ("Diabetes mellitus") | 348 | 31-08-2023 |
| 4 | ScienceDirect | ("Prothrombin time" OR "Activated partial thromboplastin time" OR " Coagulation parameters" OR "Coagulation profile" OR "Hemostatic profile") AND ("Diabetes mellitus") | 228 | 31-08-2023 |
| 5. | Google scholar | (“Prothrombin time" OR "Activated partial thromboplastin time" OR " Coagulation parameters" OR “Coagulation profile" OR "Hemostatic profile") AND ("Diabetes mellitus") | 1596 | 31-08-2023 |
| 6. | Cochrane  Online | (“Prothrombin time" OR "Activated partial thromboplastin time" OR " Coagulation parameters" OR “Coagulation profile" OR "Hemostatic profile") AND ("Diabetes mellitus") | 116 | 31-08-2023 |
| 7. | African journals  Online | (“Prothrombin time" OR "Activated partial thromboplastin time" OR " Coagulation parameters" OR “Coagulation profile" OR "Hemostatic profile") AND ("Diabetes mellitus") | 213 | 31-08-2023 |
| 8. | Other sources | Utilize the titles that were discovered from the reference lists of articles chosen during electronic database searches. | 10 | 2-5-09-2023 |

**Searching strategy for the evaluation of prothrombin time and activated partial thromboplastin time among diabetic and healthy controls in Africa: Systematic Review and Meta-analysis.**
